# Computer Administered Safety Planning for Individuals at Risk for Suicide: Development and Usability Testing

**DOI:** 10.2196/jmir.6816

**Published:** 2017-05-15

**Authors:** Edwin D Boudreaux, Gregory K Brown, Barbara Stanley, Rajani S Sadasivam, Carlos A Camargo, Ivan W Miller

**Affiliations:** ^1^ University of Massachusetts Medical School Worcester, MA United States; ^2^ Perelman School of Medicine of the University of Pennsylvania Philadelphia, PA United States; ^3^ Department of Psychiatry Columbia University College of Physicians & Surgeons and New York State Psychiatric Institute New York City, NY United States; ^4^ Massachusetts General Hospital Boston, MA United States; ^5^ Butler Hospital and the Warren Alpert Medical School of Brown University Providence, RI United States

**Keywords:** technology, safety, health planning, suicide, computers, telemedicine

## Abstract

**Background:**

Safety planning is a brief intervention that has become an accepted practice in many clinical settings to help prevent suicide. Even though it is quick compared to other approaches, it frequently requires 20 min or more to complete, which can impede adoption. A self-administered, Web-based safety planning application could potentially reduce clinician time, help promote standardization and quality, and provide enhanced ability to share the created plan.

**Objective:**

The aim of this study was to design, build, and test the usability of a Web-based, self-administered safety planning application.

**Methods:**

We employed a user-centered software design strategy led by a multidisciplinary team. The application was tested for usability with a target sample of suicidal patients. Detailed observations, structured usability ratings, and Think Aloud procedures were used. Suicidal ideation intensity and perceived ability to cope were assessed pre-post engagement with the Web application.

**Results:**

A total of 30 participants were enrolled. Usability ratings were generally strong, and all patients successfully built a safety plan. However, the completeness of the safety plan varied. The mean number of steps completed was 5.5 (SD 0.9) out of 6, with 90% (27/30) of participants completing at least 5 steps and 67% (20/30) completing all 6 steps. Some safety planning steps were viewed as inapplicable to some individuals. Some confusion in instructions led to modifications to improve understandability of each step. Ratings of suicide intensity after completion of the application were significantly lower than preratings, pre: mean 5.11 (SD 2.9) versus post: mean 4.46 (SD 3.0), *t*_27_=2.49, *P*=.02. Ratings of ability to cope with suicidal thoughts after completion of the application were higher than preratings, with the difference approaching statistical significance, pre: mean 5.93 (SD 2.9), post: mean 6.64 (SD 2.4), *t*_27_=−2.03, *P*=.05.

**Conclusions:**

We have taken the first step toward identifying the components needed to maximize usability of a self-administered, Web-based safety planning application. Results support initial consideration of the application as an adjunct to clinical contact. This allows for the clinician or other personnel to provide clarification, when needed, to help the patient build the plan, and to help review and revise the draft.

## Introduction

### Background

Suicide and suicide attempts have increased over the past 10 years in the United States despite efforts by national organizations to reduce them [1-4]. Safety planning is a common suicide prevention tool designed to help an individual develop a plan for managing his or her suicidal thoughts. Although no universally accepted safety planning method exists, the Safety Planning Intervention [5,6] has gained widespread acceptance in the suicide prevention community and has been incorporated into numerous treatment guidelines and interventions [5-8]. The Safety Planning Intervention [5] is collaboratively built by a clinician with a patient and encourages individuals to engage in six sequential steps when feeling suicidal: (1) identify early warning signs, (2) employ internal coping strategies, (3) distract with social engagement or change of environment, (4) access suicide-protective social support, (5) seek help through crisis resources, and (6) restrict access to lethal means. The Safety Planning Intervention has a strong empirical foundation supporting each of its six steps [5], as well as evidence that it improves the average number of outpatient mental health visits for suicidal patients during the 6 months following the index emergency department (ED) visit, when compared with treatment as usual [9].

Although the Safety Planning Intervention is quick when compared with other suicide prevention interventions such as cognitive behavioral therapy, it commonly takes 20 min or more to administer [5,10-13]. Given that many health care settings, such as EDs and primary care settings, are characterized by multiple competing demands and suffer from serious time constraints on how much time can be spent with individual patients, the time it takes to build a collaborative safety plan remains an important barrier limiting adoption and implementation in clinical practice [10]. Moreover, it takes clinician training and practice to build a high quality, personalized safety plan [5]. Consequently, not having ready access to well-trained clinicians who know how to build a good safety plan is a barrier that further impedes widespread implementation in most health care settings, especially those not specifically devoted to providing behavioral health care. Novel approaches to safety planning that decrease staff burden by making the process less time-intensive, which support the systematic building of high-quality plans, and remain effective in preventing suicidal behavior are needed.

### Safety Planning

With the ubiquity of computers and Internet access in modern society, a Web-based, self-administered safety planning application could offer tremendous potential for accomplishing these objectives and improving scalability of safety planning. Supporting this premise, several mobile phone apps designed for self-administered safety planning are currently available [14-16]. However, these safety planning apps are not designed to be used as clinical tools in health care settings; as a result, they have significant limitations, including being dependent on a patient having a mobile phone, downloading the app successfully, and completing the safety planning steps, most of which require extensive text entry, during a clinical encounter. Should these barriers be resolved, review by the clinician on the patient’s phone is not practical. Moreover, there is no published data on the usability of these mobile phone safety planning apps. Thus, we designed, built, and tested the usability of a Web-based, self-administered safety planning application that could be completed on a desktop, laptop, or tablet computer. Our goal was to have the application include standardized instructions, “as needed” access to video instructions, and ready downstream access to the safety plan for Web-based editing, reprinting, and sharing with other clinicians and caregivers. We do not intend for the Web-based application to replace the clinician; rather, we expect it will make building safety plans more efficient for the clinician by reducing the total time required to build a safety plan by offloading the bulk of the orientation to the safety plan and the creation of the plan itself to the computer. The clinician will still maintain a role in reviewing the safety plan and editing it, as needed.

Before such a system can be widely promoted, the usability of the application must first be established, and the alpha version of the application adapted based on user feedback. The final system’s impact on clinical workflow, such as whether clinician efficiency improved when compared with traditional safety planning, and impact on suicide-related outcomes, such as whether suicidal behavior decreases, needs to be conducted. This paper describes the development process and initial results of the usability testing (ie, the first step). This is important, because usability can be seriously impacted by a variety of factors, even with a seemingly simple task, such as completing an online form. For example, usability can be adversely impacted by context or setting (eg, busy, distracting medical settings) and patient population (eg, mixed demographics, mixed computer literacy, medically ill, psychologically distressed). Moreover, this is the first systematic test of the transition of modalities from clinician administered to self-administered, and the team needed to validate that users could create at least the initial draft of the safety plans themselves.

## Methods

### Overview of Application Development

We employed a user-centered, iterative software design strategy. Our development was funded through the Emergency Department-Safety Assessment and Follow-Up Evaluation (ED-SAFE) [17] and overseen by one of the ED-SAFE’s Principal Investigators (EDB). The team comprised psychologists, psychiatrists, emergency physicians, nurses, informaticians, software engineers, and additional subject matter experts, including the developers of the Safety Planning Intervention [5] (GKB, BS), and included both experienced researchers and active clinicians. The entire team was intimately involved with every step of the design and testing process to ensure that the application was firmly rooted in the latest advances in safety planning and was user-friendly. It was built to be compliant with applicable health care security and privacy standards, including the Health Insurance Portability and Accountability Act (HIPAA).

**Figure 1:**
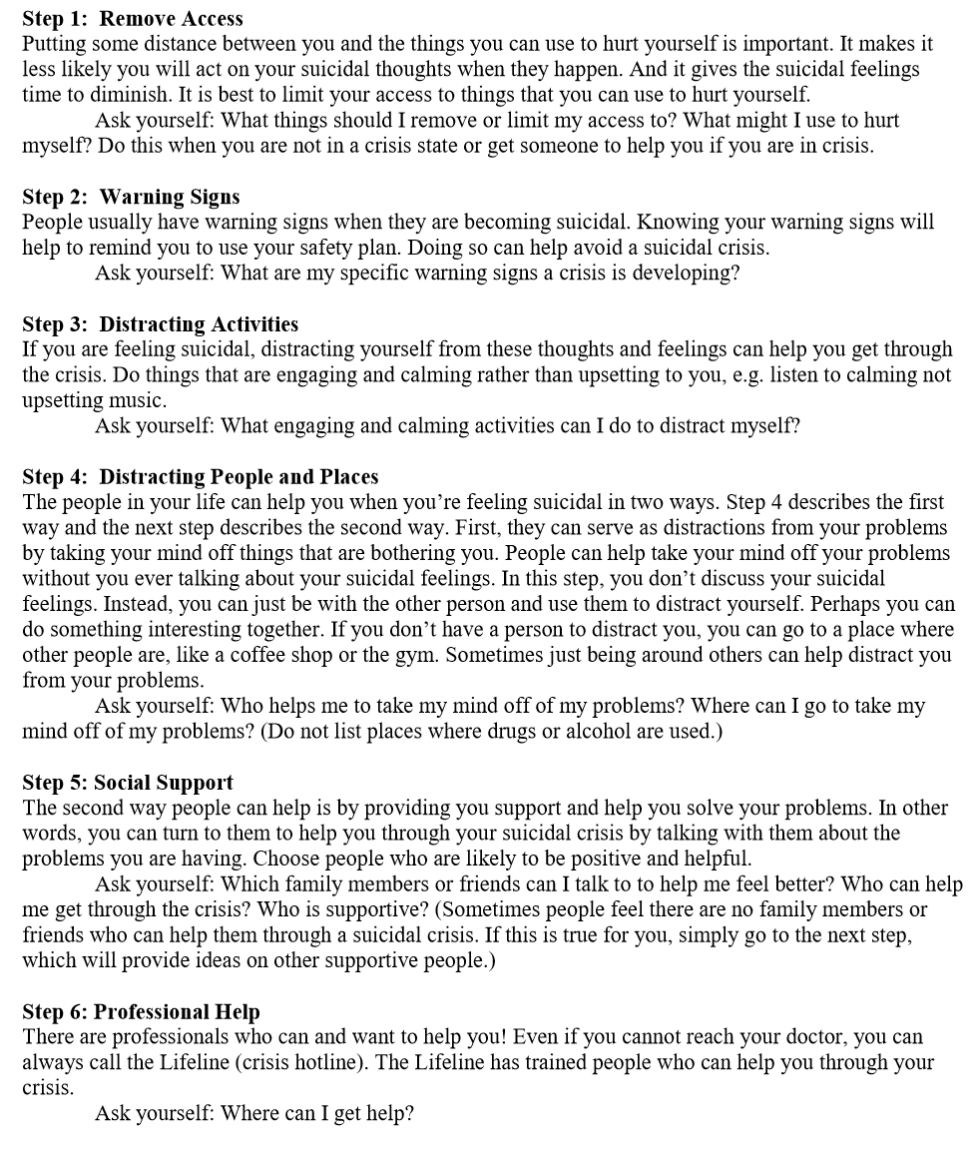
Safety planning application steps.

The computer application used the same six steps as the Safety Planning Intervention, but the order of the steps was changed to put means restriction first, rather than last, because members of the team noted that it is important for the individual to restrict means to suicide immediately upon discharge from the hospital. Presenting means restriction first aligned with the presumed order of action and priority upon discharge. The new order, along with associated instructions and response options used for the application, is presented in Figure 1. Multimedia Appendix 1 contains some representative screenshots.

The original Safety Planning Intervention, like other versions of safety planning, is designed to be completed by clinician interview, with the patient’s responses documented in text on a template paper form. We considered simply computerizing this by presenting instructions for each of the six steps followed by an open text field to allow the patient to enter his or her free-text responses. However, this was viewed by the design team to be a potential barrier because of the need for computer keyboard literacy and with potential for lengthening the time of administration. Consequently, to facilitate rapid, user-friendly completion, multiple choice options were developed for the first three steps. The response options were created by soliciting suggestions from subject matter experts, iterative review by the development team and consultants, and revision based on patient input to select the final response options and to perfect the wording. A free-text field was included for each step to allow individuals to input their own responses, if and when they desire. This combination of multiple choice and free-text balanced the need for simplicity with the goal of personally tailored planning. Each of the six traditional Safety Planning Intervention steps is presented sequentially, with easy-to-follow instructions and a “MORE” button that provides additional detail, if needed. A brief instructional video was developed to provide additional instructions to the patient, accessible as needed.

Once all the six steps have been completed, the individual reviews and “confirms” the safety plan. The printed version is formatted similar to the traditional Safety Planning Intervention and is designed to fit on a single page for ease of printing, access, and manipulation. The safety plan is stored on a secure server, and the individual can access the plan from any computer or mobile phone with Internet connection. The safety plan can be edited, printed, saved as a portable document format (PDF) and shared with others by email through a secure email service.

### Setting and Participants

The usability testing was set in an urban, tertiary care hospital in Central Massachusetts. Consecutively presenting adult patients being evaluated for an acute psychiatric emergency in either the ED or inpatient psychiatric unit were screened during research assistant (RA) shifts. Inclusion criteria included 18 years of age and endorsement of active suicidal ideation in the past two weeks. Exclusion criteria included persistent severe medical illness, severe emotional distress, cognitive insufficiency (eg, dementia, psychosis, altered consciousness), incarceration, and insurmountable language barriers.

### Usability Testing Procedures

Eligible patients, who agreed to participate, signed written informed consent. We used a “Think Aloud” protocol testing approach [18,19]; although the participants interacted with the application and completed their safety plan, they were asked to vocalize their thoughts, feelings, and opinions about each screen. Think Aloud informs how a user approaches the interface and his or her mental processes when utilizing the interface. Additionally, Morae (TechSmith version 3.2.1, 2010) usability software was used [20-22]. It allows for video and audio recordings of the subject being tested, including recording clicks, keystrokes, and other events. For our purpose, one reviewer reviewed the audio recordings and summarized the issues identified by the participant. Structured usability ratings and open-ended questions asking about pros, cons, and recommendations for improvement were obtained by the RA. All problems or difficulties encountered throughout the protocol through Think Aloud, direct observation, and patient interview were documented in detail and reviewed with the study team weekly. Software, instructions, and item wording were modified in response to feedback.

Because there was some concern that having patients engage in self-administered safety planning could actually have the paradoxical effect of increasing momentary intensity of suicidal ideation, participants provided ratings of suicidal ideation before and after engaging in the safety planning. Conversely, engaging with the application could have the effect of improving an individual’s perceived ability to cope with suicidal thoughts, so ratings of ability to cope with suicidal thoughts were obtained. The medical records of each participant were reviewed for one month before and after the enrollment date to assess acute suicide-related health care utilization.

### Measures

#### Demographics and Descriptives

Age, sex, race (white, black or African American, Asian or Pacific Islander, American Indian, Alaska Native, Aleut, Other), ethnicity (Hispanic, non-Hispanic), and insurance type (private, Medicare, Medicaid or State, other, none) were documented on all enrolled patients. In addition to demographics, we abstracted the following information from their medical record: presence of alcohol abuse (current intoxication or evidence of any problem use), intentional illegal or prescription drug misuse (current intoxication or evidence of any problem use), presence of depressed mood, disposition (discharged home, admitted to medical unit, admitted or transferred to psychiatric or substance abuse unit), and emergency physician discharge diagnosis.

#### Safety Planning Application

All patients were provided the laptop on the “home” page. They registered by creating a username and strong password and then read and responded to each of the six steps sequentially, as summarized in Figure 1.

#### Process Log

The RA completed a process log for each participant, documenting any problems noted, the solutions applied, and the outcome. Problems were categorized based on the following domains: technical failure (eg, Internet disconnection, hardware dysfunction), computer literacy (eg, mouse use, text entry), safety plan step completion (eg, trouble understanding how to respond, applicability of the step), interruptions during safety plan completion (eg, clinical care, meals, visitors), and use of proxy to help complete the plan (eg, family member or visitor).

#### Usability Ratings

All participants provided structured usability ratings (1=strongly disagree, 2=disagree, 3=neutral, 4=agree, 5=strongly agree) across a variety of specific tasks, including ability to easily move between screens, ability to understand the language of the safety planning steps, confidence in being able to create a safety plan, helpfulness of the instructional video (if watched), desire to send the safety plan to someone else by email, likelihood of using the safety plan if suicidal thoughts arise in the future, and understanding of how the safety plan can help manage suicidal thoughts. These domains were identified by the study team as tasks considered important for usability and most consistent with the ultimate goal of the application. Open-ended questions were used to assess the positive and negative impressions of the application, as well as recommendations for improvement. The ratings and interview were administered by the RA after the participant completed their safety plan.

#### Suicide Ratings

Two aspects of suicidal ideation were measured immediately before and after engagement with the application: suicidal ideation intensity (0=none, 10=constant) and perceived ability to cope with suicidal ideation (0=no ability to cope, 10=strong ability to cope). Although standardized, validated instruments measuring suicidality have advantages, there were two reasons we used simple 0-10 point items instead. First, we enrolled subjects in the ED setting. This setting is very time-sensitive, and patients were often uncomfortable or distressed, so we wanted the total participant burden to be as light as possible, precluding lengthier scales. Second, we were interested in detecting changes over a very short period of time—pre- and post-engagement with the software. Standardized scales are not designed to detect short-term state-changes in suicidal ideation.

#### Emergency Department Utilization Review

The medical records for the month before and after the index visit were reviewed. All ED visits were noted and classified based on whether or not they were related to suicidal ideation or behavior (0=no, 1=yes). Only medical records associated with the study’s health care system were accessible. Although it would have been advantageous to acquire medical records from other health systems, this proved infeasible because of patient confidentiality laws.

### Data Analysis

The primary outcomes reflecting usability were summarized using descriptive statistics, including proportions, means, and standard deviations (SDs). The safety planning application technical completion rate was defined as the proportion of participants who initiated the application and were able to complete navigation from start to end without critical technical failures, usability issues, or other interruptions resulting in an aborted encounter. The safety plan step completion rate was defined as the proportion of participants who provided at least one response for each of the six steps. This completion rate was also calculated for each of the six steps individually. Process problems were summarized as present or absent based on the categories described in “Measures—Process Log” section. Descriptive statistics were calculated on usability ratings for each domain described in “Measures—Usability Ratings” section. Additional analyses included paired sample *t* test comparing suicidal ideation intensity and ability to cope with suicidal ideation pre-post safety plan administration, as well as a paired sample *t* test comparing suicide-related acute health care visits in the one month before and after the index visit. Statistical Package for the Social Sciences 22 (IBM, Armonk, NY) was used for all analyses.

## Results

### Demographics and Descriptives

A total of 69 patients with suicidal ideation or a suicide attempt were approached by the RA; of these, 37 met all of the eligibility criteria, and 81% of those eligible (30/37) consented to participate and were enrolled. The average age of the sample was 39 years old (SD 14 years), with 47% (14/30) being male, 83% (25/30) indicating white race, and 10% (3/30) indicating Hispanic ethnicity. The majority (n=25/30; 83%) were insured by Medicaid or another state program. The clinical characteristics of the sample were as follows: presence of alcohol abuse (current intoxication or evidence of any problem use), 30% (9/30); intentional illegal or prescription drug misuse (current intoxication or evidence of any problem use), 30% (9/30); presence of depressed mood, 53% (16/30); and disposition, 10% (3/30); discharged home; admitted to medical unit, 3% (1/30); and admitted or transferred to psychiatric or substance abuse unit, 87% (26/30). The most common primary emergency physician discharge diagnosis was mood-related (eg, depression, emotional distress, emotional crisis), which was assigned to 60% (18/30). The remaining had a variety of medical and substance abuse diagnoses.

### Usability

Multimedia Appendix 2 summarizes the usability statistics. All participants successfully registered, viewed all six of the safety planning build screens, and created a final, one-page safety plan PDF that could be printed, saved, or shared by secure email. However, completeness of the safety plan varied. The mean number of steps completed was 5.5 (SD 0.9), with 90% (27/30) completing at least 5 steps and 67% (20/30) completing all six steps.

It was found that 3 of the first 5 subjects experienced some type of technical failure, including interrupted Internet connection and “frozen” screen resulting from a glitch in the Morae usability software. At the time of enrollment, these problems were rectified by the RA, and they did not prevent the participants from resuming the application and completing their safety plans. Root causes were identified and addressed, resulting in none of the final 25 enrollees experiencing technical failures.

In total, 40% (12/30) of the participants reported or demonstrated at least one problem related to computer literacy, including unfamiliarity with using the mousepad and with typing on a keyboard. This did not result in any aborted safety plans, but it did require some modest technical assistance from the RA on occasion. It was found that 23% (7/30) of the participants reported or demonstrated problems with the actual process of building the safety plan that was not related to general computer literacy. The initial registration process caused some confusion because it required creation of a strong password; instructions around this process were improved, resulting in the final 20 enrollees reporting no problems with the registration process. Some participants needed clarification on how to complete individual steps. Most commonly, early in the study, patients confused Steps 4 and 5, because both involve identifying people who can help the individual in some way. Although Step 4 instructs the individual to identify people who can help *distract* from suicidal thoughts, Step 5 instructs the individual to identify people they can confide in and who can actually help them manage their suicidal thoughts and associated problems. Some of the confusion arose because the same set of people applied to both steps. This overlap is acceptable for building a safety plan, but the instructions initially were unclear, creating uncertainty within the participants. On the basis of the participant feedback, the instructions were modified to help clarify this distinction between the two steps. Instructions were improved, resulting in no difficulty identified with comprehension for the final 10 enrollees.

Completion of Step 5 (*identifying individuals who can help the individual through a suicidal crisis*) demonstrated the highest incompletion rate; this was not only because of the aforementioned confusion with Step 4, but also because some participants simply did not have anyone with whom they felt comfortable talking about their suicidal thoughts. Consequently, they left it blank. This perceived lack of personal relevance was a common cause for other steps being left blank as well. For example, Step 1 (*lethal means restriction*) was not answered by an individual who reported not having any lethal means accessible to him, and Step 6 (*professional services to access when suicidal*) was left blank by an individual who had no existing physician or clinician and did not know the address of the nearest ED, so could not populate any of the fields. The software was modified to allow patients to select “skip or does not apply.”

Interruptions during the process were relatively common (n=8/30; 27%) due to testing, meals, and visitors. These interruptions did not prevent the individuals from building a safety plan, but it often required them to pause the process and resume later. Although the instruction video was available to all, only 17% (5/30) chose to watch it, with the others stating that they did not think they needed to because the text instructions were clear.

In general, the usability ratings were strong, with the averages ranging from 3.8 (*helpfulness of the video; n=5*) to 4.4 (*understanding safety plan step instructions*). It was found that 80% (24/30) of the participants agreed or strongly agreed that they would use their safety plan in the future if they experienced suicidal ideation. Open-ended comments generally supported the favorable usability.

### Suicide Ratings

Ratings of suicide intensity after completion of the application were significantly lower than preratings, pre: mean 5.11 (SD 2.9) versus post: mean 4.46 (SD 3.0), *t*_27_=2.49, *P*=.02. Ratings of ability to cope with suicidal thoughts after completion of the application were higher than preratings, with the difference approaching statistical significance, pre: mean 5.93 (SD 2.9), post: mean 6.64 (SD 2.4), *t*_27_=−2.03, *P*=.05.

### Suicide Related Acute Health Care

The total number of ED visits related to suicide decreased from an average of 1.00 (SD 0.45) visit in the month before the index visit to an average of 0.07 (SD 0.25) after the index visit, *t*_29_=11.34, *P*<.001. In the month before the index visit, 93% (28/30) of patients had one or more suicide-related ED visits; in the month after the index visit, 7% (2/30) of participants had one or more suicide-related ED visits.

## Discussion

### Principal Findings

We designed and built a self-administered safety planning application for use with patients at risk for suicide and assessed its usability in 30 suicidal patients. All 30 participants were able to successfully navigate the software and produce a safety plan. Self-reported usability ratings were generally positive, even with early recruits using the initial version that had not yet benefited from adjustments resulting from user testing. However, our observations revealed important parameters surrounding the application’s usability and optimal deployment, including the need for technical problem solving during early deployment, ensuring the availability of clinical or other personnel to help with usability issues if and when they arise, and review and editing of the safety plan with a clinician, with particular attention given to understanding skipped steps.

We experienced technical problems in 3 of the first 5 participants. These were navigated in real-time by the RA, allowing the 3 participants to complete the safety plan, and root cause analyses reduced technical failures to 0 for the final 25 enrollees. This experience highlighted the importance of identifying and addressing rudimentary technical issues before and during initial clinical deployment. Despite generally positive self-reported usability ratings, 40% (12/30) of participants self-reported or demonstrated upon direct observation at least one minor computer literacy issue. None of these computer literacy problems prevented the individual from building a safety plan; instead, it simply caused the build to take longer than it would have otherwise taken if the issues had not been encountered and, at times, resulted in the RA having to provide instruction, such as showing the individual how to use the mousepad.

It was found that 33% (10/30) participants skipped at least one of the six steps, which was due to two primary reasons. First, some of the instructions were not clear about exactly what the step required of the individual. Most of the confusion centered around Steps 4 and 5, as described in the “Results” section. Both Steps 4 and 5 required the participant to identify people who can help the individual in some way, with Step 4 identifying people who can help distract from suicidal thoughts and Step 5 identifying people they can confide in and who can actually help them manage their suicidal thoughts and associated problems. This led to confusion because many individuals did not understand the distinction, or pointed out that the same set of people applied to both steps. With improvements to the clarity of the instructions, these issues were resolved. Second, individual steps that were not viewed as applicable or personally relevant, were skipped. This occurred most frequently with Step 5, which many patients chose to skip because they had no one in their social network with whom they felt comfortable divulging their suicidal thoughts. Reluctance to discuss suicide is not uncommon; it may also be reflective of the tendency for suicidal individuals to be socially isolated [23,24]. As a result, the software was modified to allow an individual to indicate if he or she desired to skip the step. It is important for clinicians to review skipped steps with the patient to determine the reason and to help problem-solve alternatives. Safety planning is primarily a process of helping patients to learn to use coping strategies during a suicidal crisis. We believe clinicians, who are competently trained in this intervention, are an essential component. However, the degree of clinical involvement required when using the safety planning Web application could be minimized and streamlined. The two models—traditional safety planning administered by a clinician using a paper-based form versus safety planning facilitated by the self-administered, Web-based application with clinician review—warrants further study in a randomized clinical trial examining not only clinical efficacy on suicide-related outcomes but also workflow efficiency, cost effectiveness, and clinician and patient satisfaction.

The application allowed multiple choice and free-text options for the first three steps. All participants completed the first three steps, with the vast majority choosing at least one of the multiple choice response options for each step. Every multiple choice response option across the three steps was selected by at least one participant. Free-text responses were entered by many as well, ranging from 23% (7/30) for Step 3 to 53% (16/30) for Step 1. This pattern supports continued use of multiple choice options, their relevance supported by their selection by patients, as well as inclusion of the free-text capabilities to allow for added personalization.

Engagement in the application did not appear to increase suicidal ideation intensity; in fact, the pattern was the opposite. Statistical decreases in suicidal ideation intensity, and increases in perceived ability to cope with suicidal ideation, were observed when comparing pre- and post-ratings. ED visits related to suicidal ideation or behavior also decreased in the month after enrollment, when compared with the number of visits in the month before enrollment. These indices were exploratory and interpretation should be conservative, because there was no control condition against which to compare performance. Moreover, decreases in ED visits after the index visit could be the result of other interventions delivered to mitigate the participants’ suicide risk, such as inpatient psychiatric hospitalization, which occurred in 87% (26/30) participants.

### Limitations

The safety planning application is subject to the limitations inherent in a Web-based platform; it requires a computer, Internet connectivity, some computer literacy, and general literacy. The use of multiple choice options may funnel patients into options that are not tailored to them, a scenario that the option of free-text fields may only partly counteract. The study was a usability study, not a trial, so it did not follow patients prospectively or have a control condition against which to compare the application’s impact on suicidal ideation or behavior. Furthermore, although a self-administered version has scalability, it may lack the personalization and expert guidance that a clinician-administered version has. For example, the clinician-administered version aids patients in determining which strategies and individuals will be most helpful and safest for them, allowing for a collaborative evaluation of their value, a task that patients, on their own, may not be able to accomplish. Another limitation was the sample size. It was small and may not represent all suicidal patients. This is acceptable for early usability tests; further testing in a large-scale trial should also seek to better identify subpopulations for whom the intervention is most appropriate and effective. Finally, the RA attempted to preserve naturalistic, real-world administration. However, she was present to observe and document problems and answer any questions. This is an inevitable tension in early usability studies that are also designed to obtain information to improve the software through using real-time feedback.

### Conclusions

A computerized, self-administered safety planning system that produces high-quality safety plans anywhere, anytime is highly innovative. Although most medical settings are usually fraught with pressing time demands for clinicians, leading to deprioritizing interventions like safety planning, patients often have considerable downtime as they are waiting for tests, clinicians, consultations, or procedures. Many settings simply lack the available interventionists to provide safety planning during the encounter. Our application has strong potential to address this problem. However, before its full impact can be tested, establishing its usability in challenging real-world settings is an essential first step. We have built the application with downstream dissemination in mind and have taken the first step toward identifying the components needed to maximize usability and foster adoption in clinical settings. It is not intended to be a stand-alone intervention. Rather, it is intended to allow the patient to begin the safety plan and allows for the clinician or other personnel to provide clarification, when needed, to help the patient build the plan, and to help review and revise the draft. A deployment where the patient can ask questions and receive technical assistance, whereas having clinicians inquire about skipped steps to clarify the reasons for the omission, is optimal. The next step is to demonstrate clinical efficacy of this model.

## Appendix

Multimedia Appendix 1 Safety planning intervention Web application steps 1-5 screenshots.

Multimedia Appendix 2 Usability statistics (N=30).

## Abbreviations

ED: emergency department
ED-SAFE: Emergency Department-Safety Assessment and Follow-Up Evaluation
HIPAA: Health Insurance Portability and Accountability Act
PDF: portable document format
RA: research assistant
SD: standard deviation
