# Supplementary material for: Computer Administered Safety Planning for Individuals at Risk for Suicide: Development and Usability Testing
Source: J Med Internet Res. 2017 May 15;19(5):e149. doi: 10.2196/jmir.6816 (PMC5447822; doi:10.2196/jmir.6816)
Supplement: Multimedia Appendix 1 [file jmir_v19i5e149_app1.pdf]

Create (be sure to click the bottom "Done" button of the last (Step 6) page when completed)

Step 1: Remove Access

Putting some distance between you and the things you can use to hurt yourself is important. It makes it less likely you will act on your suicidal thoughts when they happen. And it gives the suicidal feelings time to diminish. It is best to limit your access to things that you can use to hurt yourself.

Ask yourself: *What things should I remove or limit my access to? What might I use to hurt myself?* Do this when you are not in a crisis state or get someone to help you if you are in crisis. *('HELPFUL HINTS')*

- [ ] Get rid of pills I don't need; keep only quantities that are not dangerous.
  - My doctor, pharmacist, or other healthcare professionals, can advise me.
  - Have someone else hold my pills.
- [ ] Temporarily store all guns with a friend, relative, gun shop, or storage facility; ask someone to hold on to the keys to my gun locks / gun safe.

Other actions to limit ways I can hurt myself: ________

<< Back | Next >>

Cancel (discard changes)

Keep Myself Safe

Welcome, Test! [Log Off]

Please note that logging on as a registered user, your permanent Safety Plan is *saved with us*. It will be available for any future downloading, emailing, and on-site references or updates.

Home | MY SAFETY PLAN | Values Review | View Instructions | Tools | Suicide Lifeline | About

Step 2: Warning Signs

People usually have warning signs or triggers when they are becoming suicidal. Knowing your warning signs will help to remind you to use your safety plan. Doing so can help avoid a suicidal crisis.

Ask yourself: *What are my specific triggers or warning signs a crisis is developing?* *('HELPFUL HINTS')*

☐ Feeling down, sad, or crying	☐ Feeling alone or rejected	☐ Withdrawing, feeling isolated
☐ Feeling hopeless things won't ever get better	☐ Drinking or using drugs	☐ Feeling angry, argumentative, or short-tempered
☐ Feeling worthless or a failure	☐ Feeling trapped, no way out	☐ Feeling overwhelmed by life circumstances (e.g. finances)
☐ Being in physical pain	☐ Feeling anxious, agitated	☐ Failing, doing poorly at something

Others: ________

<< Back | Next >>

Cancel (discard changes)

Keep Myself Safe

Welcome, Test! [Log Off]

Please note that logging on as a registered user, your permanent Safety Plan is *saved with us*. It will be available for any future downloading, emailing, and on-site references or updates.

Home | MY SAFETY PLAN | Values Review | View Instructions | Tools | Suicide Lifeline | About

Step 3: Distracting Activities

If you are feeling suicidal, distracting yourself from these thoughts and feelings can help you get through the crisis. Do things that are engaging and calming rather than upsetting to you, e.g. listen to calming not upsetting music.

Ask yourself: *What engaging and calming activities can I do to distract myself?* *('HELPFUL HINTS')*

☐ Watch television	☐ Read	☐ Go for a walk, exercise
☐ Do something nice for someone else	☐ Listen to music	☐ Do a hobby, favorite activity
☐ Go online, play video games	☐ Meditate, pray	
Others:		

<< Back   Next >>

Cancel (discard changes)

Keep Myself Safe

Welcome, Test! [Log Off]

Please note that logging on as a registered user, your permanent Safety Plan is *saved with us*. It will be available for any future downloading, emailing, and on-site references or updates.

Home | MY SAFETY PLAN | Values Review | View Instructions | Tools | Suicide Lifeline | About

Step 4: Distracting People and Places

The people in your life can help you when you're feeling suicidal in two ways. Step 4 describes the first way and the next step describes the second way. First, they can serve as distractions from your problems by taking your mind off things that are bothering you. People can help take your mind off your problems without you ever talking about your suicidal feelings. In this step, you don't discuss your suicidal feelings. Instead, you can just be with the other person and use them to distract yourself. Perhaps you can do something interesting together. If you don't have a person to distract you, you can go to a place where other people are, like a coffee shop or the gym. Sometimes just being around others can help distract you from your problems.

Ask yourself: *Who helps me to take my mind off of my problems? Where can I go to take my mind off of my problems?* (Do not list places where drugs or alcohol are used.) *('HELPFUL HINTS')*

Person or place:	
Person or place:	
Person or place:	

<< Back   Next >>

Cancel (discard changes)

Step 5: Social Support

The second way people can help is by providing you support and help you solve your problems. In other words, you can turn to them to help you through your suicidal crisis by talking with them about the problems you are having. Choose people who are likely to be positive and helpful.

Ask yourself: *Which family members or friends can I talk to help me feel better? Who can help me get through the crisis? Who is supportive?*

(Sometimes people feel there are no family members or friends who can help them through a suicidal crisis. If this is true for you, simply go to the next step, which will provide ideas on other supportive people.) *('HELPFUL HINTS')*

	(phone number examples: 1(123)456-7890, or 1-123-456-7890, etc.)
Name and phone number:	
Name and phone number:	
Name and phone number:	

<< Back   Next >>

Cancel (discard changes)
