# Supplementary material for: Computer Administered Safety Planning for Individuals at Risk for Suicide: Development and Usability Testing
Source: J Med Internet Res. 2017 May 15;19(5):e149. doi: 10.2196/jmir.6816 (PMC5447822; doi:10.2196/jmir.6816)
Supplement: Multimedia Appendix 2 [file jmir_v19i5e149_app2.pdf]

Domain		n (%)	Mean (SD[a])
**Safety plan completion**			
**App technical completion**			
	Completed	30 (100)	
	Aborted or critical failure	0 (0)	
**Completion of safety plan steps**			5.5 (0.9)
	Step 1: Means restriction	30 (100)	
	Step 2: Warning signs	30 (100)	
	Step 3: Distracting activities	30 (100)	
	Step 4: Distracting people and places	27 (90)	
	Step 5: Social support	21 (70)	
	Step 6: Professional help	26 (87)	
	All 6 steps	20 (67)	
**Individual entered a free-text response**			
	Step 1: Means restriction	16 (53)	
	Step 2: Warning signs	10 (33)	
	Step 3: Distracting activities	7 (23)	
	Step 4: Distracting people and places	N/A[b]	
	Step 5: Social support	N/A	
	Step 6: Professional help	N/A	
**Technical failure experienced**			
	Yes	4 (13)	
**Computer literacy problem experienced**			
	Yes	12 (40)	
**Safety planning completion problem experienced?**			
	Yes	7 (23)	
**Interruption experienced (eg, medical testing)**			
	Yes	8 (27)	
**Proxy assisted with completion**			
	Yes	2 (7)	
**Usability ratings**			
**Move between screens easily**			4.3 (0.8)
	Strongly disagree	0 (0)	
	Disagree	1 (3)	
	Neutral	3 (10)	
	Agree	12 (40)	
	Strongly agree	14 (47)	
**Understand safety planning step instructions**			4.4 (0.6)
	Strongly disagree	0 (0)	

	Disagree	0 (0)	
	Neutral	2 (7)	
	Agree	13 (43)	
	Strongly agree	15 (50)	
**Helpfulness of the video (n=5)**			3.8 (0.4)
	Strongly disagree	0 (0)	
	Disagree	0 (0)	
	Neutral	1 (20)	
	Agree	4 (80)	
	Strongly agree	0 (0)	
**Confidence in ability to use system to create or revise the safety plan**			4.3 (0.6)
	Strongly disagree	0 (0)	
	Disagree	0 (0)	
	Neutral	2 (7)	
	Agree	17 (57)	
	Strongly agree	11 (37)	
**Understand how safety plan can help manage suicidal thoughts or feelings**			4.4 (0.6)
	Strongly disagree	0 (0)	
	Disagree	0 (0)	
	Neutral	1 (3)	
	Agree	17 (57)	
	Strongly agree	12 (40)	
**Likelihood of using safety plan if has suicidal ideation**			4.0 (0.7)
	Strongly disagree	0 (0)	
	Disagree	1 (3)	
	Neutral	5 (17)	
	Agree	17 (57)	
	Strongly agree	7 (23)	

[a]SD: standard deviation.

[b]N/A: not applicable.
